# Factors Guiding the Orientation of Nymphal Spotted Lanternfly, *Lycorma delicatula*

**DOI:** 10.3390/insects14030279

**Published:** 2023-03-11

**Authors:** Miriam F. Cooperband, Jacob D. Wickham, Melissa L. Warden

**Affiliations:** 1 Forest Pest Methods Laboratory, USDA—APHIS—PPQ, 1398 W. Truck Rd., Buzzards Bay, MA 02542, USA; 2 Institute of Zoology, Chinese Academy of Sciences, 1 Beichen West Rd., Beijing 100101, China

**Keywords:** tree effects, orientation, aggregation behavior, attraction, population density, *Lycorma delicatula*, methyl salicylate lure, population distribution, dispersion

## Abstract

**Simple Summary:**

The spotted lanternfly, *Lycorma delicatula* White (Hemiptera: Fulgoridae), has recently emerged as a destructive invasive pest that is a great challenge to control. To develop and improve trapping, detection, and mitigation tools, it is crucial to understand what factors guide their behavior. Recent studies show that SLF aggregate, but the mechanisms driving aggregation behavior are poorly understood. This study evaluated the orientation behavior of SLF nymphs in the field when offered specific combinations of three factors: trees with different densities of SLF, tree size, and the presence of a semiochemical lure. When evaluated as a choice between two opposite characteristics while controlling for the two other factors, preferences were discernable, and all three factors were found to be attractive to SLF nymphs. Larger tree sizes and higher densities of SLF were highly attractive, and when forced to choose between them, only first instars revealed a preference for higher SLF density over larger-sized trees. When these two factors were controlled, methyl salicylate lures demonstrated four weeks of efficacy in the field but could not outcompete the draw of larger tree size or higher SLF density.

**Abstract:**

A mark–release–recapture experiment was conducted to evaluate the orientation of spotted lanternfly (SLF) *Lycorma delicatula* White (Hemiptera: Fulgoridae) nymphs when released equidistant between two trees. The experiment was repeated weekly for eight weeks in a heavily infested area with mature tree-of-heaven *Ailanthus altissima* (Mill.) Swingle (Sapindales: Simaroubaceae) planted in rows as ornamental street trees in Beijing, China. One tree in each pair received a methyl salicylate lure, and the lure was rotated between trees every week as it aged. Two additional independent variables for each tree were also analyzed: size and SLF population density. Marked–released SLF significantly chose trees with higher SLF population density over trees with lower density populations, and they also chose larger trees significantly more than smaller trees. Population density and tree size were better predictors of attraction than lures, but when those factors were controlled, SLF significantly chose trees with methyl salicylate lures over control trees for the first 4 weeks of lure life. Wild SLF distribution was assessed weekly, revealing strong aggregation in first and second instars that diminished with development to the third and fourth instars. Thus, nymphal SLF aggregate, and orientation is strongly guided by the presence of other SLF and tree size.

## 1. Introduction

In 2014, in Berks County, Pennsylvania, the first detection was made of the spotted lanternfly *Lycorma delicatula* (White) (Hemiptera: Fulgoridae) (SLF) in the United States. Since then, this pest has spread to numerous counties in Pennsylvania and multiple states in the northeastern United States [1]. The SLF is predicted to successfully establish in many parts of the United States, including New England, the mid-Atlantic, central, and pacific regions of the country [2,3].

The SLF is a plant hopper native to China. Its preferred host plant for feeding and oviposition, *Ailanthus altissima* (Mill.) Swingle (Simaroubaceae) [4,5,6,7,8,9] was introduced into the United States in 1784 and has since become widespread throughout much of the country, flourishing in disturbed areas [10]. Despite its preference for *A*. *altissima*, the SLF feeds on the sap of at least 103 host plant species in 33 families and 17 orders, including, but not limited to, grapes, maples, oaks, walnuts, and poplars [8,11,12]. SLF poses a serious threat to several crops and ornamentals in the United States, including grapes, stone fruits, apples, and hops [12,13,14,15]. It became an invasive pest in Korea in 2006 [16] and in Japan in 2008 [17], having been documented as a major pest of grapes and a nuisance pest in Korea [18,19,20], as well as the United States [15]. The SLF inflicts damage on plants both directly, through feeding in large numbers, and indirectly, by expelling honeydew onto surrounding plant material [14,21,22,23]. The accumulating honeydew promotes the growth of sooty mold, which inhibits photosynthesis and contaminates fruit meant for market [4,23].

**Figure 1:**
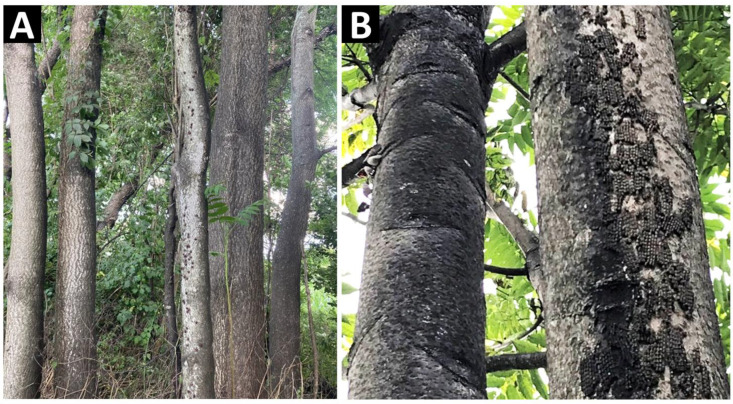
The image on the left (**A**) shows a group of *A. altissima* trees in Pennsylvania on which one tree (center) has a population of fourth instar spotted lanternflies (SLF), *L. delicatula*, but no SLF can be seen on the surrounding trees. The image on the right (**B**) shows two adjacent *A. altissima* trees in Pennsylvania, one of which (right) is coated with SLF egg masses and the other is not. (Photo credit: Kelly Murman).

SLF have been observed aggregating in large numbers on *A*. *altissima* and other hosts, and they appear to form a clumped distribution [12,24,25,26,27]. We have observed, both in China and the USA, that SLF in any stage aggregated on some trees while being apparently absent on adjacent trees (Figure 1). Although the possibility of nutritional differences between host plants has been proposed [25], other factors are likely involved in SLF arriving or arresting on one host over another and thus should be considered. Possible mechanisms driving this phenomenon could involve semiochemicals such as kairomones from host plants [9] or semiochemicals from SLF honeydew exudates [28], pheromones [29], vibroacoustic signals [30], visual cues [31,32], or some combination thereof. Despite attraction to the kairomone methyl salicylate in both laboratory and field experiments [33], numerous other kairomones that were attractive in the laboratory [9] were not attractive in the field (MFC, unpublished data) [34], and methyl salicylate also yielded inconsistent field results [35], suggesting that yet unknown factors may compete with the efficacy of those semiochemical lures in the field.

We conducted a field study using mark, release, and recapture techniques to ensure that each insect released between two trees had an equal probability of arriving on both trees in a given pair. This study used traps and lures in conjunction with a high-density naturally occurring (wild) SLF population in its native range in China. We tested the null hypotheses that equal numbers of marked–released SLF would arrive on (1) trees baited with methyl salicylate lures as compared with unbaited control trees; (2) trees with a higher density of naturally occurring SLF as compared with lower density naturally occurring SLF; and (3) trees with a larger diameter at breast height (DBH) as compared with smaller DBH.

## 2. Materials and Methods

**Figure 2:**
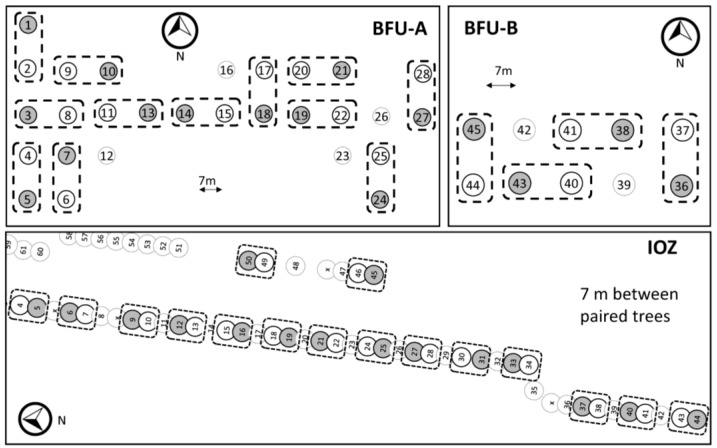
Diagrams of all *A. altissima* trees (numbered circles) present at field sites at Beijing Forestry University (BFU-A and BFU-B) and the Institute of Zoology (IOZ) in Beijing, China, in 2018. Tree pairs are surrounded with dashed lines, and the initial placement of methyl salicylate lures is indicated with gray shading. Each lure was rotated weekly to the opposing tree in the pair. The two trees in each pair were separated by approximately 7 m. A circled “x” indicates a tree that was either a different species or dead. Trees not in pairs surrounded with dashed lines were not used in this study. Note that each diagram has a different scale.

This study was conducted over eight weeks to evaluate the orientation of marked SLF nymphs given an equal probability of arriving on one of two trees, between which they were released, using a matched pair design. A total of 64 mature, evenly spaced *A. altissima* trees, in 32 pairs, were selected in urban ornamental plantings in Beijing, China, where SLF occurred naturally in relatively high densities. Two adjacent sites on the campus of Beijing Forestry University (BFU) contained 4 and 12 pairs, and the remaining 16 pairs were along a small campus street at the Institute of Zoology (IOZ), Chinese Academy of Sciences. Overall, the 64 study trees had an average and median DBH of 27.4 (±0.6 SE) cm and 28.5 cm, respectively. The average DBH difference between paired trees was 5.3 (±0.8) cm, and the distance between trees in pairs was approximately 7 m. Trees at BFU were growing roughly in a grid in an urban campus landscape with rows running N–S and E–W, and trees at IOZ were street trees growing in long rows running approximately NNE–SSW. At IOZ, there was at least one buffer tree between each tree pair (Figure 2).

This study commenced on 29 April and concluded on 25 June 2018. Every week, each tree received a fresh sticky tree band (23 cm wide, TB50M-2159; Web-Cote Industries, Inc., Hamburg, NJ, USA), which was attached at breast height. At that time, the Web-Cote sticky tree band was the most efficacious tool available for trapping SLF [32,33]. One of the trees in each pair was baited with a kairomone lure containing methyl salicylate (with an initial release rate during the first week of 54 ± 3 mg/d; details below), while the other tree in the pair had no lure. We previously demonstrated that methyl salicylate attracts all mobile stages of SLF [9,33]. Lures were placed near the top of the sticky band. Half of the methyl salicylate lures were manufactured by AgBio (Predalure MS90, Westminster, CO, USA), and the other half were manufactured by Alpha Scents (Spotted Lantern Fly Lure, West Linn, OR, USA), and both lures had comparable initial release rates. To test how long it would take for lures to become depleted or lose efficacy, the same lures were used throughout all eight weeks. In three previous SLF studies [33], methyl salicylate lures produced significant behavioral activity in the field: (1) wild second instar SLF responded in a significant dose–response fashion to methyl salicylate lures on *A. altissima* trees at the current study location, (2) third instar SLF had a significant dose–response to methyl salicylate lures placed on uniformly planted chinaberry trees, *Melia azedarach* L. (Sapindales: Meliaceae), in sites with low SLF density, and (3) both marked–released and unmarked free-living adult SLF selected *A. altissima* trees baited with the methyl salicylate lure significantly more than the paired control trees in Beijing after 9 days [33]; however, in those studies, the lures were not rotated between trees over time. In the current study, the position of the methyl salicylate lure and its paired control was rotated every week in an attempt to control for unknown effects associated with individual trees.

Each week, after new sticky bands were placed on trees, 20 marked SLF were released on the ground halfway between each pair of trees. Each tree pair that was within 7 m of another pair received a different color of marked SLF so that their release position was known. A previous field study found that nearly 96% of marked–released SLF were recaptured less than 7 m from their release point (MFC, unpublished) [36]. Each group of 20 SLF had been captured the same day and dusted with fluorescent powder (red, blue, yellow, Shannon Luminous Materials, Inc., Bioquip, Rancho Dominguez, CA, USA; Green 21WGDP, The Cary Company, Addison, IL, USA; A-21Corona Magenta^TM^, DayGlo, Cleveland, OH, USA). Based on these colors, only the SLF recaptured on the pair of trees between which they were released were included in the analysis because they started with an equal chance of being captured on those two trees. The first instars were released in weeks 1 and 2, the second instars were released in weeks 3 and 4, and so on. Captured marked and unmarked SLF on bands were tallied at the end of each week when the bands were replaced. For each tree, the number and density (unmarked SLF per cm circumference of the tree x sticky band width) of wild SLF trapped and the number of marked–released–recaptured SLF were recorded weekly. The DBH of each tree was also recorded.

Two approaches were used to evaluate where marked SLF oriented. The first explored all parameters and interactions in a single model (described below), and the second analyzed the specific choices made by marked SLF when offered precise combinations of parameters. Since the former analysis found only two significant parameters (DBH and wild SLF density), in the latter approach, in order to determine how SLF responded when these parameters were offered in different combinations, the choices of marked SLF were examined using chi-square tests on groups of paired trees for which the two trees had specific combinations of these parameters relative to one another. For instance, if the DBH of two trees in a given pair differed no more than 3 cm, they were considered similar in size (larger DBH divided by smaller DBH ranged from 1.01 to 1.09), otherwise, one was considered bigger and the other was smaller (larger DBH divided by smaller DBH ranged from 1.12 to 2). These size designations were the same each week. Conversely, wild SLF density on each tree changed every week as SLF populations moved, so relative density was determined every week for each tree pair. If the larger density divided by the smaller density in the same pair was between 1 and 1.65, then the trees in that pair were considered to have similar SLF density. Similar-density tree pairs also included one pair from week 7 in which both trees had zero SLF. Otherwise, the multiplier was 1.7 or more (up to 33.5), in which one tree in the pair was labeled as having a higher SLF population density and the other a lower density, including 13 instances over 8 weeks in which one tree in a pair had no SLF and the other had SLF. Consequently, each week the 32 tree pairs were re-categorized into the following five study groups based on their relative sizes and SLF densities: (A) size differed, density was the same, (B) size was the same, density differed, (C) one tree was bigger and had higher density and the other was smaller and had lower density, (D) one tree was bigger and had lower density and the other was smaller and had higher density, and (E) both trees had the same size and density, in which case the presence of the lure was evaluated.

The spatial pattern, or dispersion, of the wild SLF population was also examined at each site over time to determine if wild SLF trapped on sampled trees were aggregated or not.

*Statistics*. The relationships between all factors were first examined by fitting a generalized linear mixed model with a negative binomial distribution, where marked insect counts were modeled with fixed effects for treatment (lure or control), lure manufacturer, DBH, the density of wild SLF, and week, plus their interaction, a random effect for tree pair, and an offset for the number of days the traps were out. This was conducted using the lme4 package [37] in R (R Core [38]). In the second analysis, the frequencies of marked SLF choices were compared using a chi-square goodness-of-fit test with the null hypothesis that marked–released SLF would arrive at each tree in the pair with equal frequency. A significant choice occurred at α = 0.05 when the test statistic G was greater than or equal to 3.841 [39,40]. Finally, linear regression models were used to evaluate variance/mean ratios over time for each site (JMP, version 10.0.0, SAS Institute, Cary, NC, USA). 

To evaluate the weekly spatial dispersion pattern of the wild population at each site, three different indices were calculated, each with different advantages and limitations [41]. The first approach is the variance-to-mean ratio or Dispersion Index (*I*), one of the most commonly used and reliable estimates of dispersion [41]. By simply dividing the variance by the mean, the resulting index specifies whether a population is distributed in a uniform manner (*I* < 1), randomly (*I* = 1), or is contagious (aggregated) (*I* > 1) [41,42,43]. Its drawbacks are that it primarily describes Poisson-distributed populations, and it is affected by the sample size and density [41,44]. The second approach is Morisita’s Index of Dispersion (*I_d_*) [44], which somewhat eases these limitations, but is also affected by sample size and population density to some extent [41]. Finally, the more complex Standardized Morisita Index (*I_p_*), addresses most of these issues, limits the range of results to between −1 and 1, and provides a 95% confidence interval, and is thus considered one of the best measures of dispersion [41,45]. For the latter two approaches, an index at zero indicates a random distribution, a more negative index approaches uniform distribution, and a more positive index indicates a contagious or clumped distribution. For each sampling unit or quadrat (tree), the number of wild SLF captured, and the number per tree adjusted for size (SLF per cm circumference × 25) were used to calculate dispersion indices.

## 3. Results

After tracking the relative density of the two adjacent trees in each of the 32 pairs over eight weeks, it was discovered that a dominant (higher SLF density) tree in a pair does not always stay dominant. Interestingly, of the 11 pairs of trees that started with similar densities in the first week, one pair continued to have a similar density over eight weeks, six pairs of trees flipped back and forth between which tree was dominant, and four pairs maintained one dominant tree with a higher or similar SLF density than the other. For the 21 tree pairs that started with one tree having a higher density than the other, eight pairs continued to have the same tree dominant for seven or eight weeks, seven pairs reversed which tree in the pair was dominant, and six pairs fluctuated between one tree being dominant and both trees being similar in density over the eight weeks. Thus, it appeared as though whole populations of SLF were moving and fluctuating in time and space. Therefore, treatments using relative density assignments in the choice tests did not always involve the same trees or tree pairs each week.

**Figure 3:**
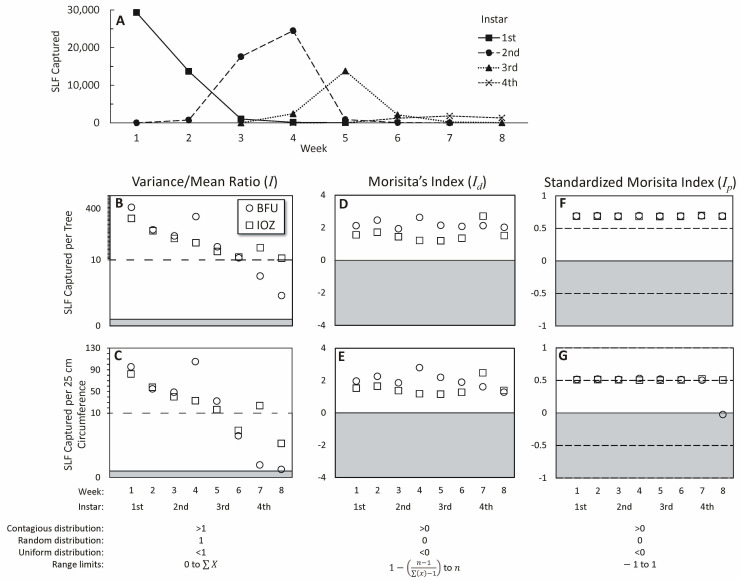
The (**A**) total number of wild (unmarked) spotted lanternflies (SLF), *L. delicatula*, captured in each stage and week are plotted. For each week and site the number of free-living SLF (*x*) captured per tree and SLF captured per tree then standardized by tree size, respectively, were used to calculate (**B**,**C**) the Variance-to-Mean Ratio (*I*), (**D**,**E**) Morisita’s Index (*I_d_*), and (**F**,**G**) Standardized Morisita’s Index (*I_p_*). At each site, Beijing Forestry University (BFU) (circles) and the Institute of Zoology (IOZ) (squares), there were 32 sampling units (trees), which are represented as *n*. Indices in the white fields represent populations with contagious (aggregated) distribution, whereas indices in the gray fields approach uniform distribution, and indices where the fields converge represent random distribution. The dashed horizontal lines for *I_p_* indicate the 95% confidence interval at 0.5 and −0.5. Distributions and limits for each index are given below [41].

The recapture rate over eight weeks was 27% for SLF recaptured on any tree, and 25% for SLF recaptured within the same tree pair they were released. The majority of each instar (unmarked) was trapped during its corresponding two-week rotation period (Figure 3A), and 65 adults were captured at the end of week 8 marking the beginning of adult eclosion. 

The dispersion index (*I*) indicated that natural populations at both field sites were highly aggregated on trees, with I values being greater than 1 for all weeks (ranging from 410.3 to 4.6), but the degree of aggregation diminished significantly over the 8 weeks and four instars (linear regression for BFU and IOZ, respectively, R^2^ = 0.7832, 0.8408; *p* = 0.004, 0.001) (Figure 3B). This reduction in *I* values corresponded with reduced total captures of larger SLF instars on sticky bands. When corrected for tree size, aggregation was still found to occur among all stages, but the fourth instars showed a distribution approaching random at BFU, where only 3.4 SLF per tree were captured on average that week (Figure 3C). Morisita’s index (*I_d_*) showed that all stages at both sites were aggregated (Figure 3D), regardless of tree size (Figure 3E). The standardized Morisita index (*I_p_*) showed that populations were significantly aggregated for all stages and locations (Figure 3F), regardless of tree size, except for week 8 at BFU, when *I_p_* was −0.025 (slightly uniform) when only 3.4 SLF were captured per tree on average (Figure 3G).

**Figure 4:**
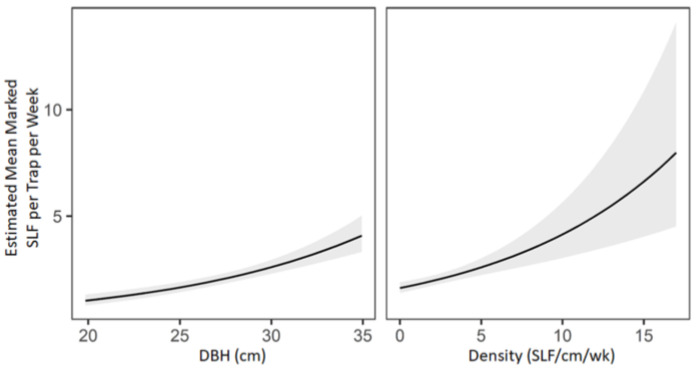
The predicted value (black line) ±95% confidence interval (gray shading) of the catch of marked spotted lanternflies (SLF), *L. delicatula*, based on tree size (DBH) and SLF density, with each graph keeping all other model covariates constant.

In the multifactorial analysis, no differences in marked SLF were found between lure manufacturers, and the number of marked SLF captured on trees with a lure versus control varied with the week of the experiment (interaction $\chi^{2}$ = 14.174, df = 7, *p* = 0.048), but no post hoc comparisons of the treatment effect of the lure by week were significant. The post hoc analysis revealed that higher counts of marked SLF were driven by the DBH of the tree (χ^2^ = 54.05, df = 1, *p* < 0.001) and the background density of SLF on the tree (χ^2^ = 22.23, df = 1, *p* < 0.001). The effects of each of those characteristics on the catch of marked SLF, while keeping all other model covariates constant, are graphed in Figure 4.

**Figure 5:**
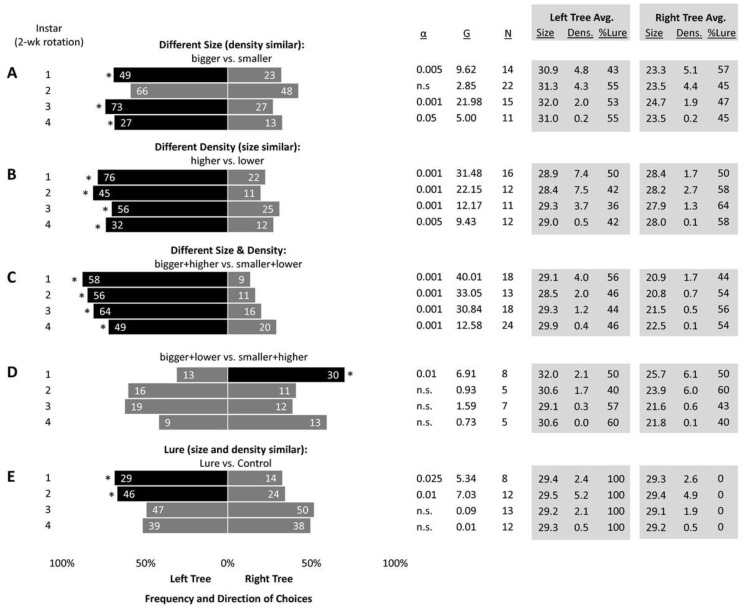
The frequency and direction of choice by marked–released–recaptured spotted lanternfly (SLF), *L. delicatula*, nymphs in four 2-week rotation periods (instars) when released weekly between 32 pairs of trees with different combinations of sizes (cm DBH), wild SLF densities (wild SLF/cm circumference), and presence of lures. Groupings of treatments contained paired trees, represented on the left and right side of the graph, respectively, that either were (**A**) different sizes (bigger vs. smaller) but had the same wild density, (**B**) the same size but had different densities (higher vs. lower), (**C**) one tree was bigger with higher density (bigger + higher) and the other tree was smaller with lower density (smaller + lower), (**D**) one tree was bigger with lower density (bigger + lower) and the other was smaller with higher density (smaller + higher), or (**E**) both trees were equal in size and density but one tree had a methyl salicylate lure (Lure) and the other had no lure (Control). Every lure was used for 8 weeks, and its position in its tree pair was rotated each week. Asterisks indicate when SLF choices deviated significantly from random, with α ≤ 0.05 when the test statistic G ≥ 3.841, and black bars indicate the direction of the significant attraction. The number of times 20 marked SLF were released between a pair of trees is shown (N), as well as the average size (cm DBH), density (SLF/cm circumference), and percent of trees with methyl salicylate lures for the left and right bars. Numbers inside bars represent the numbers of marked SLF caught. The specific tree pairs represented in each row differed each week due to fluctuations in wild SLF density.

Chi-square tests of marked SLF choices by grouped tree pairs, based on relative size and wild SLF density, revealed that first, third, and fourth instars oriented to larger trees significantly more than smaller trees when the density was the same (Figure 5A). All nymphal instars oriented significantly more to higher density trees than lower density trees when the size was the same (Figure 5B). All nymphal instars oriented significantly more to larger and higher density trees over smaller and lower density trees (Figure 5C). However, when larger trees with lower density were paired against smaller trees with higher density, forcing SLF to make a choice between size or density, only the first instars made a significant choice and were captured significantly more on the smaller trees with higher density, suggesting a higher importance of density over size for first instars (Figure 5D). When trees were equal in size and density, SLF oriented to trees with lures significantly more than control trees for the first two rotation periods, suggesting that lure efficacy lasted 4 weeks (Figure 5E), but the overall model revealed that lures alone were not able to outcompete the draw of larger trees and higher density populations.

## 4. Discussion

This mark–release–recapture experiment on *A. altissima* trees in Beijing revealed that large tree size and high background SLF population density were both significantly more likely to guide SLF orientation than methyl salicylate lures. Although the density of the wild SLF population was positively correlated with tree size, each factor independently played an important role in the orientation of SLF nymphs. Only the first instars prioritized higher SLF density over larger trees. Whether SLF moved to trees with higher background populations of SLF on them because the SLF were producing attractive signals (i.e., pheromones, substrate vibrations, or damage kairomones) [28,29,30], or because there was something initially more attractive about those trees (i.e., kairomones, visual cues, or nutritional quality) [9,31], which consequently lured both the marked and wild SLF, could not be ruled out from this experiment. However, the fact that tree size is also strongly attractive and correlated to SLF density suggests that there could be a positive feedback loop in which SLF may initially be attracted to larger trees, and once groups of SLF find those trees, the combination of the larger trees and signals from SLF may result in more SLF attraction. 

Naturally occurring SLF populations had aggregated distributions across all instars (Figure 3B,D,F), and this was independent of tree size, although more SLF did occur on larger trees (Figure 3B,C). With trees being the sampling unit, and tree sizes varying, if SLF were distributed evenly per cm^2^ of the tree, larger trees would appear to have larger numbers of SLF and appear to give a clumped distribution [44]. However, aggregation was also detected when tree size was controlled (Figure 3C,E,G). In weeks 5–8, the average numbers of captured SLF progressively diminished from hundreds per tree to only a few per tree, and lower capture rates also might have decreased the dispersion index [44]. If egg masses are deposited in aggregations, then this could enhance the effects of first instar aggregations. The naturally occurring first instar distribution would likely reflect the distribution of the egg masses from which they just emerged, but marked–released first instars clearly oriented to the higher-density trees with first instars already on them (Figure 5B,D). SLF appear to preferentially deposit egg masses on certain parts of trees over others [46]. There also exists some evidence that egg masses may be placed in aggregations favoring some trees over others [47]. We have observed large numbers of egg masses in aggregations on one tree but not on adjacent trees (Figure 1B), and the aggregation behavior of females during oviposition time has been documented [26]. First instar nymphs emerging from egg masses that are aggregated would start off with an aggregated distribution; however, this study shows that marked–released SLF nymphs oriented towards the trees with aggregations already on them.

The ability of SLF on the ground to orient to and locate the trees with the larger SLF populations, when tree size is controlled, suggests that some form of signal or communication is employed. Such signals could include semiochemicals, visual cues, or acoustic signals. A possible signal that might be detected from the ground could be honeydew falling onto surfaces beneath infested trees. Adult male SLF orient to the volatiles from conspecific honeydew [28], so it is possible that nymphs also communicate through signals derived from their honeydew, facilitating aggregation or location of suitable host plants. Host volatiles, or kairomones, released by host plants have been shown to be attractive to SLF both in the laboratory and field [9,33]. A set of specific kairomones could also be released from the host plant as a result of feeding damage, and could signal to SLF the presence of conspecifics or a suitable host to feed upon. The insects themselves could emit chemical signals such as pheromones that also could indicate the presence of conspecifics and facilitate aggregation. Evidence that pheromones may exist in SLF honeydew and body volatiles has been reported for adults as a possible mechanism for observed aggregation behavior and for mate location [28,29], and aggregation in SLF nymphs could also be mediated by pheromones. The first evidence of pheromones in the suborder Auchenorrhyncha, to which fulgorids belong, was recently reported [48], and pheromones have been documented in the two other major hemipteran suborders Heteroptera (true bugs) [49,50] and Sternorrhynca (aphids, whiteflies, psyllids, scales) [51,52,53,54]. Acoustic communication is common in other members of Hemiptera, and many utilize multimodal communication in which a combination of pheromones and substrate vibrations are employed [50]. Early evidence has been found revealing that SLF adults and nymphs can potentially communicate through substrate vibrations [30], but research aimed at understanding vibro-acoustic communication in SLF is in its infancy, and it has not been explored for other members of Fulgoridae.

## 5. Conclusions

This investigation demonstrated that SLF aggregate as nymphs, and as nymphs, SLF orientation is strongly guided by both the presence of other aggregations of SLF as well as tree size, two factors that are attractive and independent of each other. The presence of these attractive cues outcompeted methyl salicylate lures, but when differences between tree size and population density were eliminated, methyl salicylate lures showed significant efficacy for four weeks under field conditions. Our ongoing investigations seek to better define SLF communication mechanisms in order to develop and improve tools for early detection and mitigation.

## Data Availability

All data will be made available to the journal as requested.
